# Editorial

**Published:** 1863-07

**Authors:** 


### Editorial.

Mercy Hospital.—It is well known to most of our readers, and especially to those who have visited or attended the medical colleges in this city, that the Hospital of the Sisters of Mercy has afforded the chief resources for direct clinical or bed side instruction ever since its commencement in the autumn of 1850. It is equally well known, that up to the present time its prosperity and usefulness have been greatly retarded by the want of a suitable hospital building. Hence, we could make no more gratifying announcement, both to the friends of the sick and of medical education, than the fact, that during the next thirty days the hospital will be transferred to entirely new quarters. An ample plot of ground, beautifully improved, and occupied by a well-constructed building, on the corner of Calumet Avenue and Rio Grande Street, has been secured and fitted up as a permanent location for the hospital. The building is constructed of brick, 40 by 80 feet, and four storys high. It contains six public wards for ordinary medical and surgical patients, both male and female; six smaller rooms, admirably adapted for the accommodation of those patients whose circumstances render them desirous of better and more quiet rooms than can be had in a general ward; and a ward especially for lying-in women. We shall thus have a general hospital, worthy of the name, and adequate to the wants of the city. It will continue, as heretofore, under the excellent management of the Sisters of Mercy. The medical wards will continue under the charge of Prof. N. S. DAVIS; the surgical, under the charge of Prof. E. ANDREWS; and the lying-in department, under the care of Prof. W. H. BYFORD. Being located only a few blocks distant from the new building for the Medical Department of Lind University, it will continue as fully accessible for clinical instruction as heretofore.

---

MEDICAL DEPARTMENT OF LIND UNIVERSITY.—The friends of this Institution will be gratified to learn that its next Annual Course of instruction will be given in an entirely new and permanent College edifice. The latter is now in process of erection, and will be completed before the end of September next. It is located in the South Division of the City, near the corner of State Street and Ringgold Place. It is being built of brick, three storys above the basement, and will contain a Library and Dispensary Room, Laboratory, Museum, Dissecting Room, College Hall or Lower Lecture Room, and Amphitheatre. Being constructed especially for a Medical College building, it will contain all the conveniences and comforts that are desirable in such institutions. The location is directly between the Mercy Hospital and the City Hospital building, and so near that students will have easy access to both; thus enabling the Faculty to retain the most ample clinical advantages, as a part of their regular course of instruction. The present Summer Course of instruction, which is now nearly completed, has been a very pleasant and profitable one; and the institution, in all its relations, is steadily increasing in its prosperity.

---

Since the above was in type, we have received the following official announcement from the Faculty of the College, which we insert with pleasure:—

TO THE FRIENDS AND PATRONS OF THE CHICAGO MEDICAL COLLEGE,—MEDICAL DEPARTMENT OF LIND UNIVERSITY:

Since the issue of our Annual Announcement at the close of the last Lecture Term, some changes and improvements have been made, which we are sure will be gratifying to all the friends of a more systematic and extended system of medical education.

The Board of Trustees of the Lind University having determined to change the name of that institution, to that of "Lake Forest University," it became necessary to choose some other name for the Medical Department, to prevent frequent mistakes in reference to its locality. It had also become necessary for the Faculty of the Medical Department to become organized as a corporate body, to enable it to receive and hold property independent of the Board of Trustees. For these reasons, it has been deemed advisable to adopt, as a separate and permanent name, the *Chicago Medical College;* and hence, by this title the Institution will be known and designated hereafter.

An improvement of much greater importance to the friends of the school, consists in the acquisition of new and commodious college building and grounds, located in a pleasant and very accessible part of the city. The building is now advancing rapidly towards completion, and will be fully ready for occupancy at the commencement of the next regular Annual Lecture Term. In planing the building, no unnecessary expenditures were incurred for mere external architectural show; but such internal arrangements were made as to secure every needed accommodation and convenience. Its location is such as to secure a continuance also of the most ample means for hospital clinical instruction, being only a few blocks distant from the new Mercy Hospital, on the one side; and still nearer to the City Hospital building, on the other.

In thus announcing the important improvements being made in our college accommodations, it is deemed advisable to add a brief statement of the principles on which the Institution is founded, and the objects its Faculty aim to accomplish, as follows:—

First, That the Medical College should embrace such a number of Professorships, and such length of Lecture term, as will enable the Faculty to give a fair review of all the important branches of medical science during each term.

Second, That the several branches should be so grouped as to enable the student in his several courses to pass from the more fundamental to the more practical branches, as in all other departments of education.

Third, That the instruction in all the departments or branches should be demonstrative, as far as possible, and not merely theoretical.

In accordance with these principles, the founders of the Chicago Medical College adopted a curriculum of instruction embracing thirteen professorships, namely, Descriptive Anatomy; Physiology and Histology; Inorganic Chemistry; Materia Medica and Therapeutics; General Pathology and Public Hygiene; Surgical Anatomy and Operations of Surgery; Organic Chemistry and Toxicology; Medical Jurisprudence; Obstetrics and Diseases of Women and Children; Principles and Practice of Surgery and Military Surgery; Principles and Practice of Medicine; Clinical Surgery; and Clinical Medicine.

They divided these into two groups, called the junior and senior departments or courses. The five first named branches constitute the junior course; and the remaining branches the senior course.

The full Collegiate year embraces nine months, namely, from the first of October to the first of July. Five months of this period, namely, October, November, December, January, and February, constitute the regular Annual Lecture Term; and the remaining four months constitutes a summer reading and Clinical Term. The regular Lecture Season, or Winter Term, embraces full courses of instruction on all the branches named in the curriculum of both junior and senior departments. And the hours of lecturing are so arranged that any student can attend, if he chooses, the lectures on all the branches in both departments throughout the term.

But all first course students are *required* to attend, faithfully, the branches embraced in the junior course; and are required to undergo a careful examination in those branches at the close of the term. Second course students are required to attend all the branches in the senior course; while those who attend a third course, can select such branches from both departments as they may choose.

The clinical course embraces a regular medical or surgical clinic in the hospital and dispensary, one hour each day, throughout the Collegiate year.

The Summer Term of four months embraces one familiar lecture and one clinic each day.

The system of medical college instruction, thus arranged, has a comprehensiveness equal to the present extended range of medical sciences; a systematic order of progress, favorable alike to thorough acquisition of knowledge and desirable mental discipline; while the daily hospital clinics render the courses on the practical branches as demonstrative as those on the more elementary.

Both for the purpose of interesting the profession of the State more directly in the cause of medical education, and for avoiding all cavil in reference to the character of the final examination of candidates for graduation, the Faculty have requested the Illinois State Medical Society to appoint annually a Board of three Censors to attend on, and participate in, the examinations at the close of each Annual Course of instruction.

The system thus devised has now been in practical operation four years; and its success has been more than equal to the expectations of the Faculty.

Hence we again invite the attention of the profession to it, in connection with the new College Building, with a full conviction that it will meet with universal favor. The following constitute the present active members of the Faculty:—

J. S. Jewell, M.D., Professor of Descriptive Anatomy.

H. A. Johnson, M.D., Professor of Physiology and Histology.

J. H. Hollister, M.D., Professor of Materia Medica and Therapeutics.

Henry Wing, M.D., Professor of General Pathology and Public Hygiene.

F. Mahla, Ph. D., Professor of Inorganic Chemistry.

Edmund Andrews, M.D., Professor of Principles and Practice of Surgery, and of Military Surgery.

Ralph N. Isham, M.D., Professor of Surgical Anatomy and Operations of Surgery.

W. H. Byford, M.D., Professor of Obstetrics and Diseases of Women and Children.

N. S. Davis, M.D., Professor of Principles and Practice of Medicine, and of Clinical Medicine.

F. Mahla, Ph. D., Professor of Organic Chemistry and Toxicology.

H. G. Spafford, Professor of Medical Jurisprudence.

J. S. Jewell, M.D., Demonstrator of Anatomy.

The regular Lecture Term commences on the second Monday in October, and continues until the first Tuesday in March, following.

The Summer Term commences on the second Tuesday in March, and continues until the first Monday in July.

The fees are as follows:—

| | |
|---|---|
| For the Winter Term, admitting to all the Lectures in the College, | $50.00 |
| Graduation Fee, | 20.00 |
| Marticulation Fee, | 5.00 |
| Dissecting Ticket, | 5.00 |
| Hospital Ticket, | 6.00 |

All fees are payable in advance.

Summer Course free to matriculated students of the College.

For further information inquire of

E. ANDREWS, *Secretary.*

Chicago, July 27, 1863.

---

Report on the Sanitary Condition of the West Division of the City. By H. Wanzer, M.D., Chicago. Read to the Chicago Medical Society, June 13th, 1863.—Dr. Wanzer represents the health of the West Division of the city as having been good during the month of May and early part of June. Yet, he says, cases of fever, chiefly of the malarious origin, have been met with, which he describes in general terms as follows:—

"For several days previous to the call of the physician, the patients usually complained of lassitude, drowsiness, headache, backache, bitter taste in the mouth, sometimes vomiting of bilious matter. These symptoms were, generally, followed by a chill." In their treatment, mercurial alteratives have been indispensable at the commencement of the attacks, followed by anti-periodics. Recoveries were, generally, rapid and complete. In relation to typhoid fever, the reporter says, "There have been but few cases of typhoid fever, though, in consequence of the malarial atmosphere we have had through the winter, and the filthy condition of the streets, alleys, &c., throughout large portions of the city, we may expect many cases later in the season."

He represents an unusual prevalence of exanthematous fevers, more especially of scarlatina, many cases of which were of the anginose variety. The milder cases uniformly recovered with but little medication. A few cases were represented to have assumed a malignant form, two of which were described nearly as follows:—

The first was a girl of six years of age. The attack was sudden and accompanied by so much swelling of the tonsils and glands of the neck as to render deglutition very difficult. At the commencement, the febrile excitement was very high, though the characteristic eruption on the skin was slight. The mouth was dry; and dark sordes gathered on the lips and gums; and, in a short time, an aplastic exudation covered the tonsils, pharynx, and some parts of the mouth, as in diphtheria. The strength rapidly failed, with a small and frequent pulse. The patient, however, recovered. The second case was a boy 3 years old. The eruption was not full, and the symptoms were of a typhoid character from the beginning; the pulse was very frequent and weak; the edges of the lips and gums became covered with dark sordes early; white exudations appeared in the mouth and fauces; the parotid regions were swollen; and, in the advanced stage of the disease, a large abscess formed in one of these regions. In their treatment, a mild aperient was first given, and followed by sulphate of quinine and muriated tincture of iron, with chlorate of potassa in solution as a gargle. Beef-tea and milk were relied upon for nourishment.

The reporter had observed only a few cases of erysipelas, two of which proved fatal. One of the latter was a child only 12 days old. The erysipelatous inflammation commenced in the right breast, and spread so rapidly that the child soon became exhausted to a fatal degree. The other fatal case was a child 5 years of age. The child had received a severe fracture of the frontal bone, with laceration of the dura mater. The depressed portions of bone had been removed by an operation; and, in twelve hours afterwards, the wound was attacked with erysipelatous inflammation, which spread rapidly over the face, scalp, and meninges of the brain, and produced death in about seventy hours after the injury. He further stated, that during the preceding few weeks, a majority of his surgical patients had shown a tendency to traumatic erysipelas. Other physicians, with whom he had conversed, had noticed the existence of the same predisposition or diathesis.

He had also seen some cases of puerpural fever during the preceding month. Hooping-cough had also been sufficiently prevalent to merit the name of a mild epidemic.

---

### CALOMEL AND TARTAR EMETIC AS REMEDIAL AGENTS.

---

Surgeon-General's Office,
Washington City, *June 12th*, 1863.

Dear Sir:—Desiring to obtain the opinions of the more eminent members of the Medical Profession relative to the indiscriminate use of Calomel and Tartarized Antimony, I have the honor to request that you will answer the following questions:

1st. To what extent do you prescribe Calomel and Tartar Emetic in your practice?

2d. Do you regard these agents as indispensable in the treatment of disease?

3d. In view of the facts that a large number of the Medical Officers of the Army are young and inexperienced, and that soldiers cannot in the field be placed beyond the influence of atmospheric vicissitudes and exposure whilst undergoing medical treatment, would you recommend that the medicines in question be issued to Army Medical Officers, except, as at present, upon special requisition?

4th. Do you or do you not think that more harm than good has resulted from the use of Calomel and Tartar Emetic as medicines?

It should be stated that the following mercurials are at present on the Supply-Table, viz.:—

Hydrargyri chloridum corrosivum; Hydrargyri iodidum flavum; Hydrargyri oxidum rubrum; Hydrargyri pilulæ; Hydrargyri unguentum; Hydrargyri nitratis unguentum; Pilulæ catharticæ compositæ; and that it is provided by paragraph 13, of Circular No. 7, dated Surgeon-General's Office, May 7, 1863, which contains the Supply-Table, and which refers to the manner of obtaining medical supplies, that "it is not the design of the Department to confine the Medical Officers absolutely to that table, either in variety or quality, but only to establish a standard for their guidance in making requisitions for supplies, leaving individual preferences to be indulged at the discretion of the Medical Director or the Surgeon-General. Neither is it supposed that the quantities of the table will always meet the necessities of unusual emergencies, as, during epidemics, or in unhealthy seasons and localities; and Medical Officers who allow their supplies to be exhausted through any such contingencies, without timely notice of their approaching necessities, will be held to a strict accountability."

I am, sir, very respectfully,
Your obedient servant,
WILLIAM A. HAMMOND,
*Surgeon-General U.S.A.*

We copy the above Circular of the Surgeon-General of the United States Army, from the *American Medical Times*, of July 4th, 1863. From the language of the Circular and from the accompanying comments of the editor, it would appear that it is addressed to "the more eminent members of the medical profession," without reference to their connection with the Medical Staff of the Army. But two weeks later, we are assured, by the editor of the same journal, that the questions in the Circular were designed exclusively for the consideration of the Medical Officers of the Army, and not for medical men in civil practice however *eminent* they might be.

This Circular is certainly a very singular one. To appreciate its peculiar bearings, it must be remembered that in May preceding the same medical officer issued his noted Order, No. 6, prohibiting the further supply of Calomel and Tartar Emetic to the Medical Staff of the Army. That order professed to be founded on the fact, that Calomel had been so abused by Medical Officers of the Army, as to have produced "not only *innumerable cases of profuse salivation*, but the *not infrequent occurrence of mercurial gangrene;*" coupled with the further allegation, "that *modern pathology has proved the impropriety of the use of mercury* in very many of those diseases in which it was formerly unfailingly administered." For these reasons, the order contained the following positive and unqualified directions, viz.:—"It is directed that it (Calomel,) be struck from the supply-table, and that *no further requisitions* for this medicine be approved by Medical Directors." It is well known that the first of the reasons assigned in the order, containing as it does a sweeping charge of mal-practice against the Medical Officers of the Army, induced severe criticism, and, in some very respectable quarters, positive denials of the truth of the charge, thus making a direct issue with the Surgeon-General in relation to the facts alleged. Under such circumstances, we certainly had reason to expect that the Surgeon-General would promptly sustain the truth of the allegations in his order, by publishing the actual *statistics of* "Salivation" and "Mercurial Gangrene," specifying the hospitals and regiments in which they occurred; and thereby not only silence further cavil in regard to the facts, but enable the profession to distinguish between the innocent and the guilty Medical Officers. But, instead of this, after a delay of two months, we have the puerile and very ridiculous Circular of Inquiry, quoted at the head of this article. We say, very *ridiculous*, for how else can we characterize the following?—

"Desiring to obtain the opinions of the more eminent members of the medical profession relative to the *indiscriminate* use of Calomel," &c. Is there any man of ordinary intelligence in the profession who could have any other than an *unfavorable* opinion of the "indiscriminate use" of Calomel, or any other medicine whatever? Why ask for "opinions" relative to a proposition stated in such form as to admit of but *one* opinion? But does the Surgeon-General, in thus artfully using the word

"indiscriminate," really persist in the desire to have the world believe the Medical Staff of the Army, over which he presides, to be composed of men incapable of any *discrimination?* Again, look at question number two:—"Do you regard these agents (Calomel and Tartar Emetic,) as *indispensable* in the treatment of disease?" Of course not. For that physician who could not select, from the other preparations of *mercury* and the large classes of alteratives and sedative nauseants in the *Materia Medica*, such articles as would enable him to treat disease successfully without the particular agents named, must certainly be possessed of very limited mental resources. But because a particular remedy may not be absolutely *indispensable*, does that constitute a good reason why its use should be prohibited? Mankind have lived and transacted all their necessary business with the aid of tallow-candles merely, therefore, *gas-lights* cannot be claimed as "indispensable."

The implied logic of the Surgeon-General would require the prohibition of gas, lest some blunderhead should chance to blow up a metre, now and then; and so we are to understand that the "*innumerable* cases of profuse salivation and the not infrequent occurrence of *mercurial gangrene*," have dwindled down to the simple pettifogging questions of this Circular; and the "Modern Pathology" which had "proved the *impropriety* of the use of *mercury*," &c., meant only *Calomel*, while the use of "Hydrargyri chloridum corrosivum; Hydrargyri iodidum flavum; Hydrargyri pilulæ; Hydrargyri unguentum," &c., are exempt from any such *impropriety*. Now, if the Surgeon-General will condescend to tell us what special mischief a "young and inexperienced" Medical Officer of the Army can do with Calomel that he cannot do equally well with blue mass, corrosive sublimate, iodide of mercury, and mercurial ointment, we will engage to tell him the exact difference between tweedledum and tweedle-dee.

---

STATISTICS OF THE CHICAGO EYE AND EAR INFIRMARY.—*By E. L. Holmes, M.D., Attending-Surgeon.*—That during the year, ending May 1, 1862, *three hundred and ninety-seven* patients, and during the year, ending May 1, 1863, *two hun-*

*dred and forty-seven* patients were under treatment, making an aggregate of *one thousand two hundred and twenty-four* that have been treated since the opening of the Infirmary in 1858.

The following is a classified list of the forms of disease which have been under treatment during the past two years:—

DISEASES OF THE EYE.

| | | | |
|---|---|---|---|
| Wounds and injuries, | 34 | Amaurosis, | 15 |
| Conjunctivitis, simple, | 43 | Cystic Tumors of Lids, | 6 |
| " granular, | 144 | Obstruction of Nasal Duct, | 13 |
| " neonatorum, | 24 | Trichiasis, | 18 |
| " scrofulous, | 46 | Inflammation of Lids, | 17 |
| " purulent, | 16 | Strabismus, | 3 |
| Ulcer of Cornea, | 17 | Cataract, | 12 |
| Opacity of Cornea, | 18 | Entropion, | 12 |
| Staphlyoma of Cornea, | 14 | Extropion, | 9 |
| Foreign Bodies on Cornea, | 7 | Asthenopœa, | 13 |
| Abscess of Upper Lid, | 6 | Pterygium, | 6 |
| Iritis, | 15 | Hydrophthalmos, | 3 |
| Occlusion of Pupil, | 9 | Obliteration of lachrymal canals, | 2 |
| Total, | | | 522 |

DISEASES OF THE EAR.

| | | | |
|---|---|---|---|
| Foreign Bodies in Ext. Meatus, | 8 | Impacted Cerumen, | 8 |
| Otorrhœa, | 26 | Thickening Membrana Tympani, | 9 |
| Polypus, | 4 | Inflammatiou of Membrana Tympani, | 18 |
| Tinnitus, | 3 | Unclassified, | 37 |
| Perforation Membrana Tympani, | 9 | | |
| Total, | | | 122 |

Of the aggregate number treated, viz., 644, *four hundred and fifty-one* were natives of foreign countries, and *one hundred and ninety-three* of the United States.

---

THE PITCHER PLANT IN SMALL POX.—*To the Editor of the American Medical Times, N. Y.*—Monday, May 18, 1863, was called to W. C., a young man 23 years of age, strong and vigorous constitution. Found him with all the premonitory symptoms of variola, the lumbar pains being particularly prominent. He had been exposed to that disease eight or ten days before. Does not remember ever having been vaccinated.

Tuesday, 19th.—Fever higher, and pain more severe; eruption beginning to appear. I gave him the usual treatment; but without entering into details, suffice to say that on Saturday 23d, there was a copious eruption of pustules about the size of small split peas, diffused over the whole body, particularly on the hands and face. The latter was so swollen as almost to close the eyes; the eruption being so thick even at this stage, as to look like one great pustule. There had been more or less delirium during the night, and the severe lumbar pains were undiminished. It now occurred to me to give the sarracenia purpurea a trial, as it was growing in abundance in a marsh near the house. I sent out and procured some of the roots, and directed the nurse to give a teacup two-thirds full of the decoction every four hours.

Sunday evening, 24th, saw him again, had been delirious the night before, but was now calm, pulse slow, skin cool, and many of the pustules shrivelling. From this time the disease never advanced, but all the pustules dried up without maturing or leaving any pitting. The root in this case had cut short the disease. Let other physicians then give a trial and report on its results. Yours, &c.,

SAMUEL MITCHELL, M.D.

*Cameron Mills, June 23d,* 1863.—*American Med. Times.*

We call attention particularly to the above case, on account of the pitcher plants growing wild throughout Canada, and the facility therefore with which every physician can try it for himself. The effect of this remedy is one of the great controversies of the day in Great Britain, where it has been sent from Nova Scotia, and administered in the small pox hospitals to some of the most severe cases, and its powers denied. We shall be happy, therefore, to hear from any physician who gives it a trial; and also to learn the localities in which it is found most abundantly.—*Canada Lancet.*

---

CHRONIC ECZEMA.—M. Peters gives the following as a very successful mode of treating this disease, viz.:—

*Saline Aperient.*—℞. Sodii Chlor. ℈iij, Magnes. Chlor. ℈ij, Sodæ Sulph. ʒv, Magnes. Sulph. ℥i, Aquæ Oij. m. Dose, two tumblersful the first morning, and one tumblerful each on the second and third morning afterwards.

*The Lotion.*—℞. Hydrag Chlor. Cor. gr. ij, Aq. Lauro Cerasi ℥i, Spts. Rect. ʒii, Aquæ ʒvij. m. The parts to be washed with this solution three times a-day.—*Revue de Therapeutique.*

The quantity of chloride of magnesium ordered, may be readily made by adding half a-drachm of the carbonate of magnesia to two drachms of muriatic acid, previously diluted with an ounce of water. And the ounce of cherry laurel water in the lotion, by adding 15m Scheele's hydrocyanic acid to an ounce of water.—*Canada Lancet.*

On Gonorrhœal Ophthalmia. By Dr. M. H. Collins, Surgeon to the Meath Hospital and County Dublin Infirmary. —This affection,—so formidable to the surgeon to deal with, and so fatal to the usefulness of the eye,—yields with marvellous rapidity to repeated weak injections. The inflamed and œdematous conjunctiva being punctured, or snipped with the scissors if necessary, a careful student can be put beside the patient's bed, and shown how to send the contents of the syringe underneath the upper lid, from the external canthus across the eyeball. In the most acute cases, a solution of a-quarter of a grain of nitrate of silver to the ounce of distilled water should be used every ten minutes, for the first hour; after that, a half-grain solution should be injected every half-hour. If this is carefully carried out for the first twenty-four hours, the patient's eye will be quite safe. A stronger solution may then be used; and, if needful, it may be followed, in a couple of days, by Guthrie's ointment of nitrate of silver, if the villous condition of the conjunctiva should seem to require it. I have followed this plan of treatment generally, for at least nine years; and in that time I have never lost an eye from gonorrhœal ophthalmia, with one exception; in that case, the pupil in charge broke the syringe, and thinking it a matter of no importance, he waited for twenty-four hours to get it replaced; by this time the cornea had sloughed in one point, and the iris protruded. The man, however, was so fortunate as to recover, with comparatively slight injury to sight. Such surgeons and pupils as followed any of these cases have been struck with astonishment at the facility with which this formidable affection is cured. I cannot at this moment remember to whom the credit of weak injections of nitrate of silver is due; my attention was drawn to it by seeing the failure of the heroic treatment, which sacrifices nearly 50 per cent of eyes in whole or in part. I found, however, that the weak solutions were insufficient for the cure of the disease unless frequently applied.—*Dublin Quarterly Journ.*

---

Syphilis.—The following paragraph occurs in a lecture by Dr. Wilks, of Guy's Hospital, on the syphilitic affections of internal organs. It enunciates an important principle in the treatment of syphilis, which we think will at once commend itself as true to the minds of most men who have had much practical experience of the disease:—

In thinking of this subject, from a therapeutical point of view, I have long been under the impression that the value of absorbent remedies, as mercury and iodide of potassium, is in proportion to the formation of such low organizable material, and that these remedies are not curative in relation to the syphilitic poison itself; thus the failure of the iodide in secondary syphilis attended only by simple rashes on the skin, but its efficacy where pains in the bones exist, and other symptoms indicative of an inflammation of the fibrous tissues, with a tendency to the production of lymph.—*Edinburgh Medical Journal.*

---

INSANE POOR.—On the 1st of January, 1862, there were in England and Wales, not including a few parishes making no returns concerning their poor, 946,166 paupers chargeable to the Poor-rates, and of this number 34,271 were insane—namely 22,960 lunatics and 11,311 idiots; in other words, 3·62 per cent of the pauperism was ascribable to insanity, the lunatics being 2·43 per cent, and the idiots 1·19 per cent. 14,936 were males, 19,335 females. 18,318 were in county or borough lunatic asylums, 1193 in registered hospitals or licensed houses, 8603 in union or parish workhouses, 985 in lodgings or boarded out, and 5172 resided with relatives.

---

COLOCYNTH.—A gentlemen in Aylmer, Canada East, informs us, that being in a drug store and noticing the seeds in a colocynth apple, he procured a few and planted them, late in the spring, in a poor piece of ground with his potatoes. They throve well and bore fruit, a few of which ripened before being destroyed by frost. He describes the plant as resembling very much that of a water melon, and the fruit to be like oranges in size and appearance. Acting on this success, we sowed a few seeds in the open ground on the first of May last, the plants are now several inches in height, but have not yet commenced to run.—*Canada Lancet.*

---

BREAST-PIN SWALLOWED BY A CHILD.—In the *Edinburgh Medical Journal*, Thomas Annandale, Esq., F.R.C.S.E., relates the case of a child, aged three years, who swallowed a breast-pin about three inches in length, which was voided in twenty hours afterwards, the child having suffered no inconvenience.—*London Lancet.*

---

FORMER AND PRESENT MORTALITY OF LONDON.—From 1765 to 1775 the mortality of London was estimated at about one in twenty, or 5 per cent; in 1862 it was one in about forty, or less than 2½ per cent.—*London Lancet.*

Oxygen Gas.—At the last sitting of the Academy of Sciences of Munich, Baron Liebig made a very interesting communication relative to some experiments made with a new apparatus,—manufactured chiefly at the expense of the King of Bavaria,—for detecting the existence and measuring the quantity of oxygen in various bodies. The experiments, Baron Liebig stated, had proved clearly that oxygen is not only evolved from the atmosphere by plants, but also in tolerably large quantities by decomposition of water in the body of flesh-eating animals. Baron Liebig thinks that the knowledge of this fact will throw quite a new light on the hitherto but imperfectly understood processes of nutrition and digestion.—*London Lancet.*

---

Extraction of a Ball.—On Tuesday last, the seventeenth anniversary of the Battle of the Sobraom, a veteran applied at the North Staffordshire Infirmary to have an iron musket ball, or grape-shot, extracted from below the shoulder-blade, where he received it in that memorable battle with the Sikhs. The operation was successfully performed under chloroform by Mr. William Henry Folker.

---

Poisonous Ballet Dresses.—It is stated, that the first representation of a new piece has just been given at Hamburg, in which the female dancers appeared in green costumes to represent water-nymphs. The stuff of which these costumes were made contained such a quantity of arsenic that the needlewomen who made the dresses fell ill, and the dancers were attacked with violent symptoms of poisoning whilst on the stage.

---

Elaterium.—Dr. Thomas, near Philadelphia, informs us that he has been very successful in growing Elaterium plants in the open ground, by seeds sown in a sunny situation in May. He collected well-matured fruit from the plants for exhibition in the latter part of August. A few seeds dropping on the ground, outlived the winter, and grew thriftily the following spring.—*Canada Lancet.*

---

Muscular Electricity.—Ranke, the German physiologist, has published, amongst the results of his investigations into the phenomena of electric currents in the muscles, the fact that dead muscle is a much better conductor of electricity than the living muscle, because, as he judges, of the presence of certain products of decomposition which do not appear till after death.

